# Analysis of head and neck cancer scRNA-seq data identified PRDM6 promotes tumor progression by modulating immune gene expression

**DOI:** 10.3389/fimmu.2025.1596916

**Published:** 2025-08-27

**Authors:** Zhenyu Wu, Thurbu Tshering Lepcha, Dawei Zhou, Zhixian He, Guillaume N. Fiches, Youngmin Park, Jinshan He, Jianwen Chen, K. A. S. N. Shanaka, Steve Oghumu, Weiqiang Zhao, Anjun Ma, Qin Ma, Jian Zhu, Netty G. Santoso

**Affiliations:** ^1^ Department of Pathology, College of Medicine, The Ohio State University, Columbus, OH, United States; ^2^ Department of Microbiology, College of Arts and Sciences, The Ohio State University, Columbus, OH, United States; ^3^ Department of Biomedical Informatics, College of Medicine, The Ohio State University, Columbus, OH, United States; ^4^ Department of Microbial Infection and Immunity, College of Medicine, The Ohio State University, Columbus, OH, United States

**Keywords:** head and neck cancer, PRDM6, immune genes, scRNA-seq, HPV

## Abstract

Head and neck squamous cell carcinoma (HNSCC) is a biologically aggressive and heterogeneous group of cancers with limited treatment options for patients who do not respond to standard therapies. While HPV-related HNSCCs tend to show better therapeutic outcomes, we still have limited understanding of the immune mechanisms underlying these cancers. Immune-responsive genes (IRGs) have emerged as critical factors in regulating both tumor progression and immune response. Recent advances in single-cell RNA sequencing (scRNA-seq) and the development of cell-type specific regulon inference tools, such as IRIS3, have provided new insights into the tumor immune microenvironment. In this study, we leveraged the IRIS3 platform to analyze scRNA-seq data from HNSCC patient samples, identifying novel transcription factor (TF)-IRG networks that contribute to tumor proliferation and immune escape. Specifically, we identified PRDM6, a histone methyltransferase, possesses the previously unknown role in promoting tumor cell proliferation by inducing IRG expression. We further demonstrated that HPV viral oncoproteins (E6/E7) up-regulate the PRDM6 expression, which associates PRDM6 with HPV-positive HNSCC.

## Introduction

Head and neck cancers (HNCs) represent the sixth most common cancer worldwide with the majority being head and neck squamous cell carcinoma (HNSCC). HNSCC typically arise from the mucosal linings of the upper aerodigestive tract (1, 2) and can be further classified according to its originating location, including the oral cavity, oropharynx, nasal cavity, paranasal sinuses, nasopharynx, larynx, and hypopharynx. Overall, HNSCC is highly heterogeneous and biologically aggressive, often associated with high rates of recurrence and mortality, especially at the advanced stages. Major risk factors of head and neck cancers include consumption of alcohol, exposure to nicotine, and infection with high-risk HPV (HPV16, 18) (3). Currently, the standard treatment of HNSCC includes surgery and/or chemo- and radiotherapy. Interestingly, HPV-associated HNSCC tends to have better responses to treatments compared to HPV-negative (4), possibly due to its viral-related immunogenicity.

Despite of multiple anti-cancer treatment options, most of the locally advanced HNSCC cases still show poor responses with frequent recurrence. Immunotherapies have recently emerged as a promising alternative strategy to treat HNSCC considering that immune escape critically contributes to tumor initiation and progression (5). However, it is still at its early stage due to the lack of knowledge regarding the mechanism of immune regulation of HNSCC (6). Recently, it has been recognized that cancer cell-intrinsic genetic events profoundly modulate the tumor immune milieu and critically determine the outcome of immunotherapies (7). Thus, leveraging tumor-intrinsic mechanisms may represent a novel approach for augmenting cancer immunotherapies. Additionally, targeting these intrinsic pathways within tumor could offer a strategic advantage in cancer treatment as it minimizes systemic side effects from widespread, off-target immune activation.

HNSCC is known to evade immunosurveillance, a critical mechanism for controlling tumor initiation and progression. Malignant cells in HNSCC employ multiple tumor-intrinsic strategies to escape immune detection, including suppression of type I interferon (IFN) signaling (11, 12), upregulation of immune checkpoint genes (8), as well as induction of inflammatory responses (13). Understanding the transcription regulation underlying these immune evasion mechanisms is essential. In particular, identifying transcription factors (TFs) that regulate immune-related genes (IRGs) and mapping TF-IRG regulatory networks within tumor cells can provide valuable insight into tumor-specific immune dysfunction, independent of the influences from the tumor microenvironment. IRGs itself has been shown to play a prominent role in not only controlling tumor initiation and progression but also participating in immune and inflammatory responses in tumor cells (8, 9). Moreover, several IRGs have been identified as tumor suppressors in various cancers, which directly impacts tumor growth (10).

Through analysis of bulk RNA-seq datasets from The Cancer Genome Atlas (TCGA) consortium, differentially expressed IRGs and associated TF-IRG networks of HNSCC have been identified (14, 15). However, such bulk RNA-seq based analysis is unable to characterize cell type-specific IRG regulation. The recent advance of single-cell RNA sequencing (scRNA-seq) technology enables the high-throughput analysis of gene expression at the resolution of individual cells. In this study, we developed a pipeline to perform the integrative analysis of multiple HNSCC scRNA-seq datasets and further infer tumor cell-specific immune regulon through the IRIS3 (16). Results from our analysis provide a deeper understanding regarding the regulation of IRG expression in HNSCC malignant cells and the contribution of IRG dysregulation to tumor cell growth and immune response (17). Our approach revealed differentially expressed IRGs and novel TF-IRG networks in malignant epithelial cells within the HNSCC microenvironment. Notably, we identified PRDM6, a histone methyl-transferase (18, 19) previously unlinked to HNSCC, as a key regulator of immune gene expression in HNSCC tumor cells, including canonical interferon-stimulated genes (ISGs) such as ISG15 and IFITM1. Additionally, our findings demonstrate that HPV viral oncoproteins (E6/E7) induces PRDM6 expression, supporting a role of PRDM6 in the pathogenesis of HPV-positive HNSCC.

## Results

### IRIS3 analysis of HNSCC scRNA-seq data identified tumor cell-specific IRG-enriched regulons

**Figure 1:**
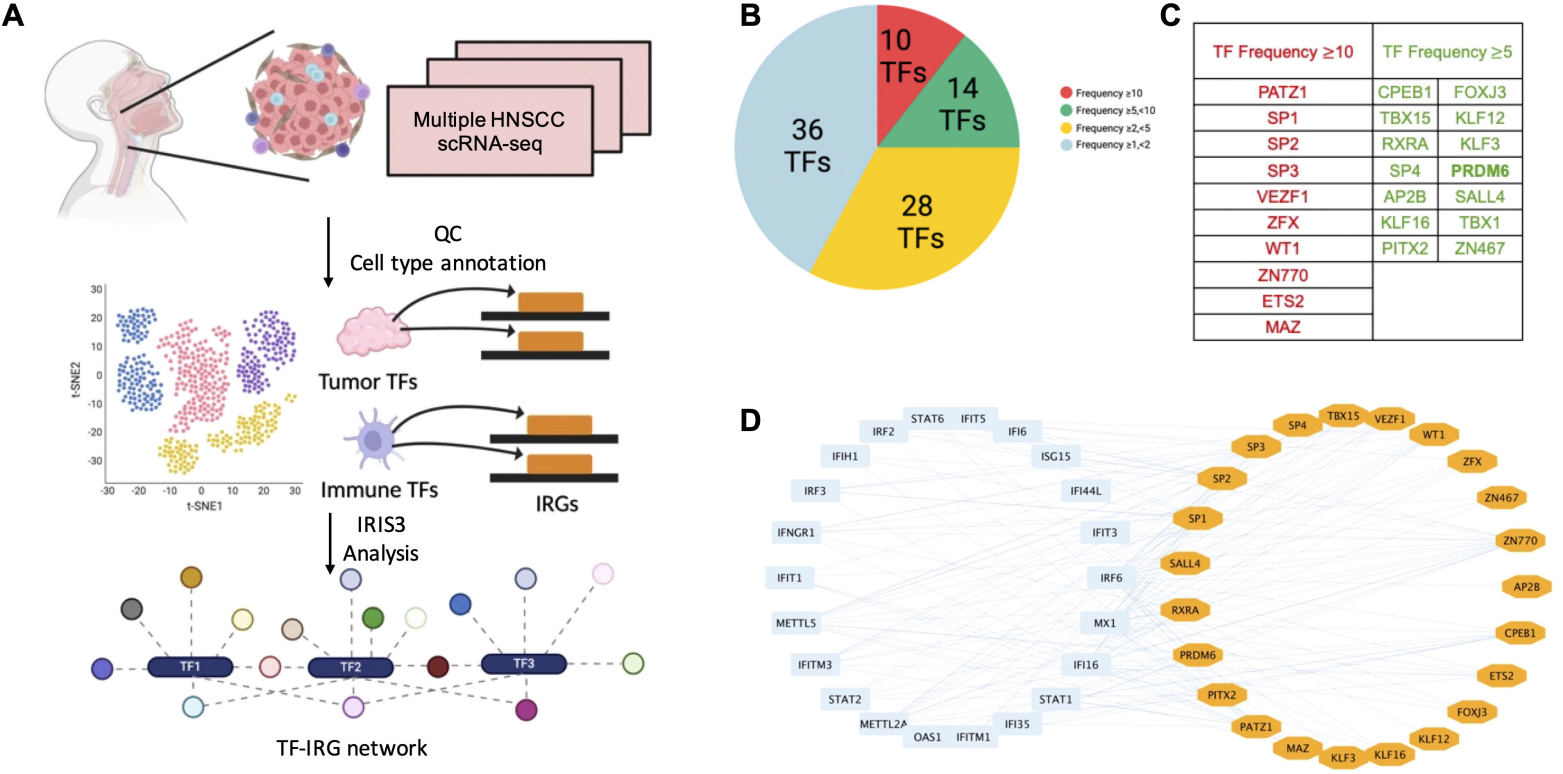
Identification of tumor cell-specific TF-IRG regulons through analysis of HNSCC scRNA-seq data. **(A)** The workflow utilized to identify tumor cell-specific TF-IRG regulons of HNSCC. HNSCC scRNA-seq datasets were collected and checked for quality. Clustering and annotation of single-cell data were performed by using Seurat. Cell-type specific regulons were identified by using the IRIS3 tool. **(B)** A total of 88 TFs were identified as regulators of IRG expression in HNSCC tumor cells. TFs are grouped according to their occurrence across multiple patients. **(C)** The table highlights all 24 TFs identified in more than 5 HNSCC patients. The left column lists 10 TFs identified in more than 10 HNSCC patients (shown in red), while middle and right columns list 14 TFs observed in more than 5 patients (shown in green). **(D)** Visualization of regulatory connections between 24 TFs (shown in orange) and selected IRGs (shown in blue) using cytoscape.

We aimed to identify tumor-specific IRG-enriched regulons by using the publicly available HNSCC scRNA-seq datasets (**Table 1**). Our pipeline consisted of initial quality-control (QC) step through Seurat to filter out low quality cells. Tumor and non-tumor cells (primarily immune and stromal cells) were annotated based on established marker genes, with existing metadata used for further validation of cell-type assignments. Datasets with a low percentage of tumor cells (less than 5%) were excluded to avoid analytical bias. In total, we included scRNA-seq data from fourteen HNSCC patients with metadata summarized in **Table 1**. To infer cell type-specific regulatory network, we applied the IRIS3 tool, which leverages public ChIP-seq data and bi-clustering methods to accurately define regulons with their cell-type specificities (**Figure 1A**). We then focused our analysis on immune regulon specifically within tumor cells, deliberately excluding immune-cell and stromal-cell derived signals to infer tumor-intrinsic regulatory network. Through this approach, we identified 639 TF-associated immune regulons that are specific for malignant epithelial cells from HNSCC tissues (**Supplementary Table S1**). To further prioritize regulons most relevant to tumor immunity, we performed the hypergeometric test to identify those that are enriched in IRGs. In total, we identified 88 tumor-specific TFs within IRG-enriched regulons that were detected in at least one patient (**Supplementary Table S2**). Among these, 10 TFs found in ≥ ten patients, 14 TFs were present in ≥ five patients, and 52 TFs appeared in ≥ two patients (**Figure 1B**). To ensure analytical stringency, we only included TFs identified in more than five patients yielding a total of 24 TFs (**Figure 1C**). Pathway analysis showed that regulons associated with these 24 TFs were predominantly involved in metabolic regulation and RNA processing (**Supplementary Figures S1A–C**). After applying IRG filtering, we observed clear enrichment of immune pathways for these regulons, including antiviral responses and the NF-κB signaling (**Supplementary Figures S1A–C**). Above all, several of the 24 TFs have been previously linked to HNSCC (20–24) (**Figure 1D**), which supports the robustness of our IRIS3-based analysis.

**Table 1:** The list of HNSCC scRNA-seq datasets used in the study.

Data Source	Cancer Type	Tumor Cells number	Tumor cells Ratio (>5%)	Patient ID
GSE103322	OSCC	2215	37.5%	N/A
GSE150430	NPC	1834	82.7%	P01
		92	28.8%	P04
		170	9.2%	P05
		233	10.5%	P06
		1975	76.3%	P11
		1069	55.9%	P12
		1501	59.5%	P13
		214	8.6%	P14
GSE162025	NPC	480	5.0%	P02
		487	7.5%	P13
		1132	15.4%	P16
GSE150321	LSCC	5777	54.0%	P01
		1321	34.0%	P02

The number and percentage of tumor cells were provided for each of 14 HNSCC patients. Certain patients were excluded from the analysis due to either insufficient number of tumor cells or low percentage.

### PRDM6 was recognized as a novel HNSCC-associated TF that regulates expression of IRGs in tumor cells

**Figure 2:**
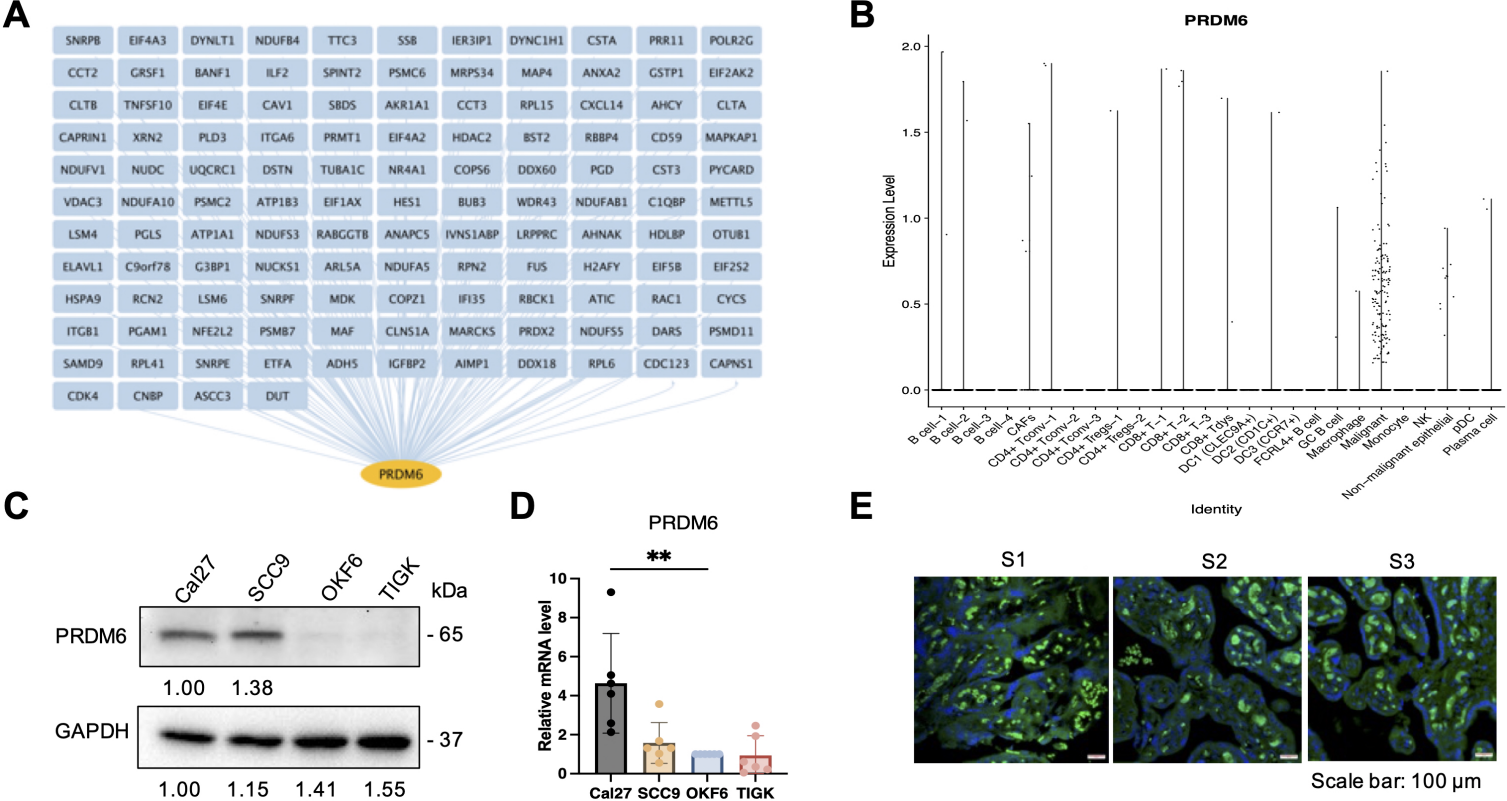
Characterization of PRDM6 as a HSNCC tumor cell-specific TF that regulates IRG expression. **(A)** PRDM6-IRG regulon identified by using the IRIS3 tool (IRG: blue; PRDM6: orange). **(B)** Expression level of PRDM6 across multiple cell types in HNSCC tumors from the public scRNA-seq dataset (GSE103322). Tumor and immune cells were annotated based on the expression of marker genes and internal labels. **(C)** Protein expression of PRDM6 in HNSCC cancer cell lines (CAL27, SCC9) as well as telomerase-immortalized normal human oral keratinocytes (OKF6/TERT-2, TIGK) was measured by protein immunoblotting analysis. GAPDH was used as the loading control. **(D)** mRNAs extracted from the above cells **(C)** were subjected to RT-qPCR analysis of PRDM6 transcript with normalization to GAPDH. **(E)** Expression of PRDM6 in tissue microarrays of three HNSCC subjects (S1-S3) was measured by protein immunofluorescence analysis with nuclei stained with Hoechst (PRDM6: green; nuclei: blue; scale bar: 100 μm). Results were calculated from two independent experiments and presented as mean ± SD. (*p < 0.05, **p < 0.01, ***p < 0.001, ****p < 0.0001, Student’s *t* test).

We focused on PRDM6 for further characterization since it has never been investigated in HNSCC. PRDM6 is a histone methyltransferase known to regulate the methylation of H3K27 (19) and H4K20 (25). Through our analysis, we identified 571 genes (listed in **Supplementary Table S3**), including 125 IRGs (**Figure 2A**), as part of the PRDM6-associated regulon. To better understand PRDM6’s role in IRG regulation, we examined its global chromatin occupancy by re-analyzing publicly available ChIP-seq datasets for human PRDM6 (**Supplementary Figures S2A, B**). We observed that the peak distributions of PRDM6 are consistent across two independent ChIP-seq datasets, GSE76496 (26) and GSE106058 (27). PRDM6 peak distribution includes introns and distal intergenic regions and approximately 14% of peaks also located near promoter regions. Additionally, we re-analyzed the CUT&RUN data of PRDM6 (GSE243557) (19), which showed a greater proportion of PRDM6 binding near promoter regions (**Supplementary Figure S2C**). This dataset also included H3K27me3 CUT&RUN profiling with PRDM6 overexpression. Our re-analysis showed that H3K27me3 peak distribution (obtained from CUT&RUN) closely mirrored PRDM6 binding patterns (obtained from ChIP-seq), supporting the notion that PRDM6 functions as a H3K27me3 methyltransferase (**Supplementary Figure S2D**). To further assess the functional impact of PRDM6, we analyzed transcriptomic changes associated with its overexpression in human neuroepithelial stem cells (GSE243554). We used DESeq2 to identify differentially expressed genes in response to PRDM6 overexpression. Subsequent pathway enrichment analysis using ClusterProfiler revealed that these DEGs are involved in the pathways related to viral life cycle and developmental processes (**Supplementary Figures S2E, F**). We further performed scRNA-seq analysis that showed PRDM6 expression is highly restricted to malignant cells and largely absent from other cell types within the HNSCC tumors (**Figure 2B**). We validated these observations by analyzing PRDM6 expression in HNSCC cancer cell lines and tissue microarrays (TMAs). Our results showed that both protein and mRNA level of PRDM6 is markedly elevated in HNSCC cancer cell lines (CAL27, SCC9) compared to telomerase-immortalized normal human oral keratinocytes (OKF6/TERT-2, TIGK) (**Figures 2C, D**). Additionally, immunofluorescence staining of TMA from three HNSCC subjects (S1-S3) confirmed the presence of PRDM6 protein within tumor tissues (**Figure 2E**). Overall, our results demonstrate that PRDM6 is a tumor-associated TF preferentially expressed in malignant cells of HNSCC.

### PRDM6 promoted cell proliferation while suppressing immune gene expression of HNSCC tumor cells

**Figure 3:**
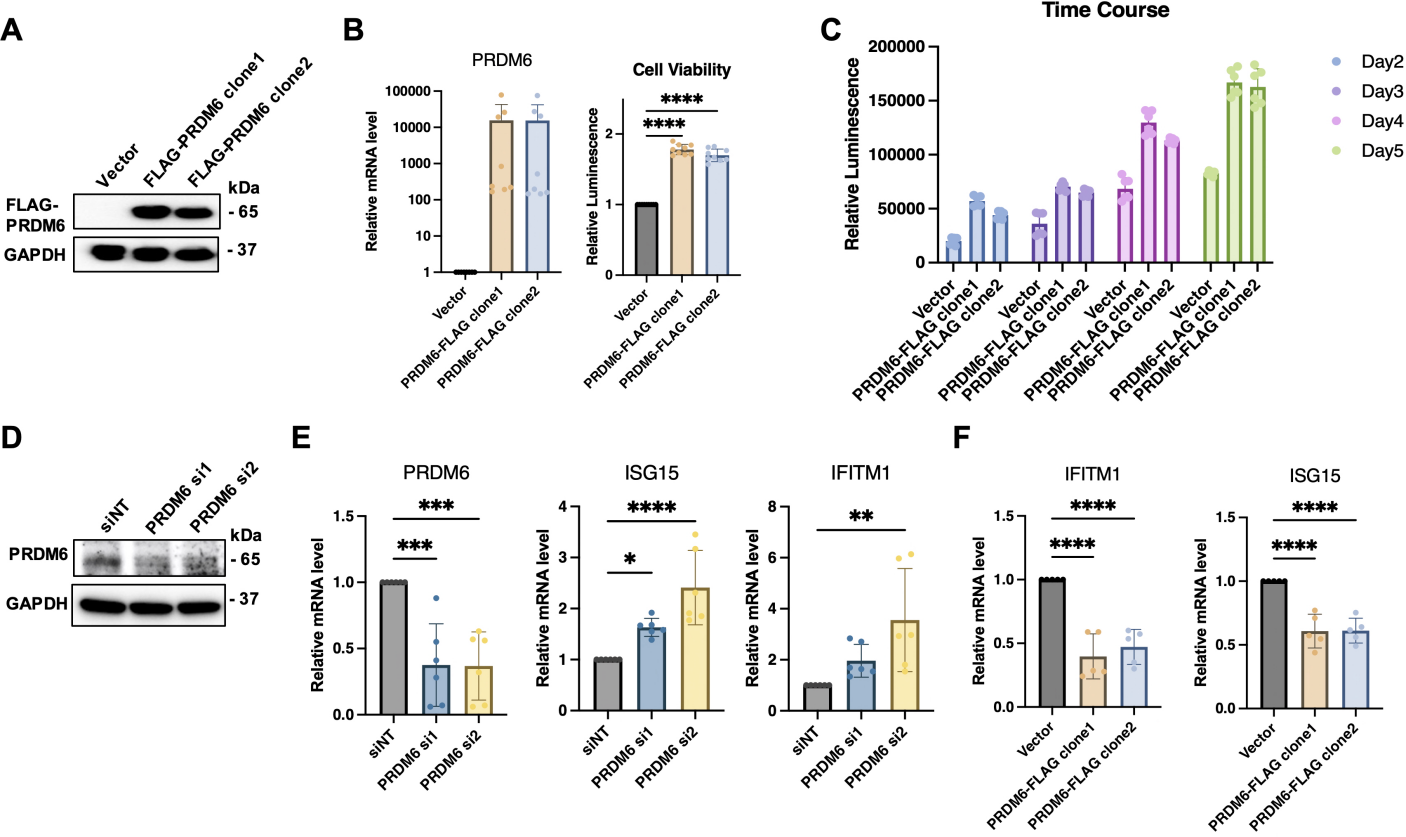
Functions of PRDM6 to promote growth of HNSCC tumor cells and suppress expression of anti-tumor ISGs. **(A)** The pcDNA vector expressing FLAG-PRDM6 was transfected in CAL27 cells to generate PRDM6-overexpressing cell clones (1 and 2). The empty vector (EV) was also transfected in CAL27 cells to generate a negative control. **(B)** A portion of the generated cell clones were harvested for RNA extraction and RT-qPCR analysis of PRDM6 transcripts with normalization to GAPDH (left panel). The remaining cells was subjected to ATP-based cell viability analysis (right panel). **(C)** CAL27 cells transfected with the pcDNA vector expressing FLAG-PRDM6 (clones 1 and 2) or EV were cultured continuously. A portion of cells was harvested at the indicated timepoints and subjected to cell viability analysis. **(D)** siRNAs targeting endogenous PRDM6 (si1 and si2) or non-targeting control (siNT) were transfected in CAL27 cells. **(E)** Total RNA was extracted and analyzed by RT-qPCR for the expression of PRDM6 or ISG transcripts (ISG15, IFITM1), with normalization to GAPDH. **(F)** CAL27 cells transfected with the pcDNA vector expressing FLAG-PRDM6 (clones 1 and 2) or EV were subjected to RNA extractions and RT-qPCR analysis of ISG transcripts (ISG15, IFITM1) with normalization to GAPDH. Results were calculated from two independent experiments and shown as mean ± SD. (*p < 0.05, **p < 0.01, ***p < 0.001, ****p < 0.0001, Student’s *t* test).

As PRDM6 was shown to express in HNSCC tumor cells, we next determined whether PRDM6 contributes to their growth *in vitro*. We cloned the human PRDM6 cDNA in the pcDNA3.1 vector, which was transfected in CAL27 to generate cell clones. Our results revealed that the PRDM6- overexpressing CAL27 cell clones (1 and 2) (**Figure 3A**) exhibit increased cell viability and proliferation compared to vector only control, as measured by cellular ATP levels (**Figure 3B**). Such effects persisted in PRDM6-overexpressing cells for up to 5 days of continuous culture (**Figure 3C**). We postulated that PRDM6 contributes to HNSCC tumor cell growth likely through dysregulation of immune gene expression based on our findings that PRDM6 is a HNSCC-associated TF that participates in the TF-IRG regulons identified from IRIS3 analysis. First, we observed that the expression of PRDM6 was moderately upregulated in CAL27 cells with IFN-α stimulation (**Supplementary Figure S3**). Second, depletion of endogenous PRDM6 by siRNA knockdown (**Figure 3C**) induced the expression of anti-tumor ISGs, ISG15 and IFITM1 (**Figure 3D**). ISG15 was previously reported to inhibit the growth of tumor cells (28, 29) while inducing their cell death (30) by targeting the NF-kB and p53 signaling. The loss of IFITM1 was also shown to induce the cell cycle arrest (31, 32). Third, we also showed that PRDM6 overexpression consistently results in the decrease of ISG15 and IFITM1 expression in CAL27 cell clones (1 and 2) (**Figure 3E**). Overall, these results indicate that PRDM6 may participate in the type I IFN signaling and control the expression of antitumor ISGs in HNSCC tumor cells, thus promoting their proliferation and growth.

### PRDM6 expression was upregulated by HPV-16 E6/E7 viral oncoproteins in HNSCC tumor cells

**Figure 4:**
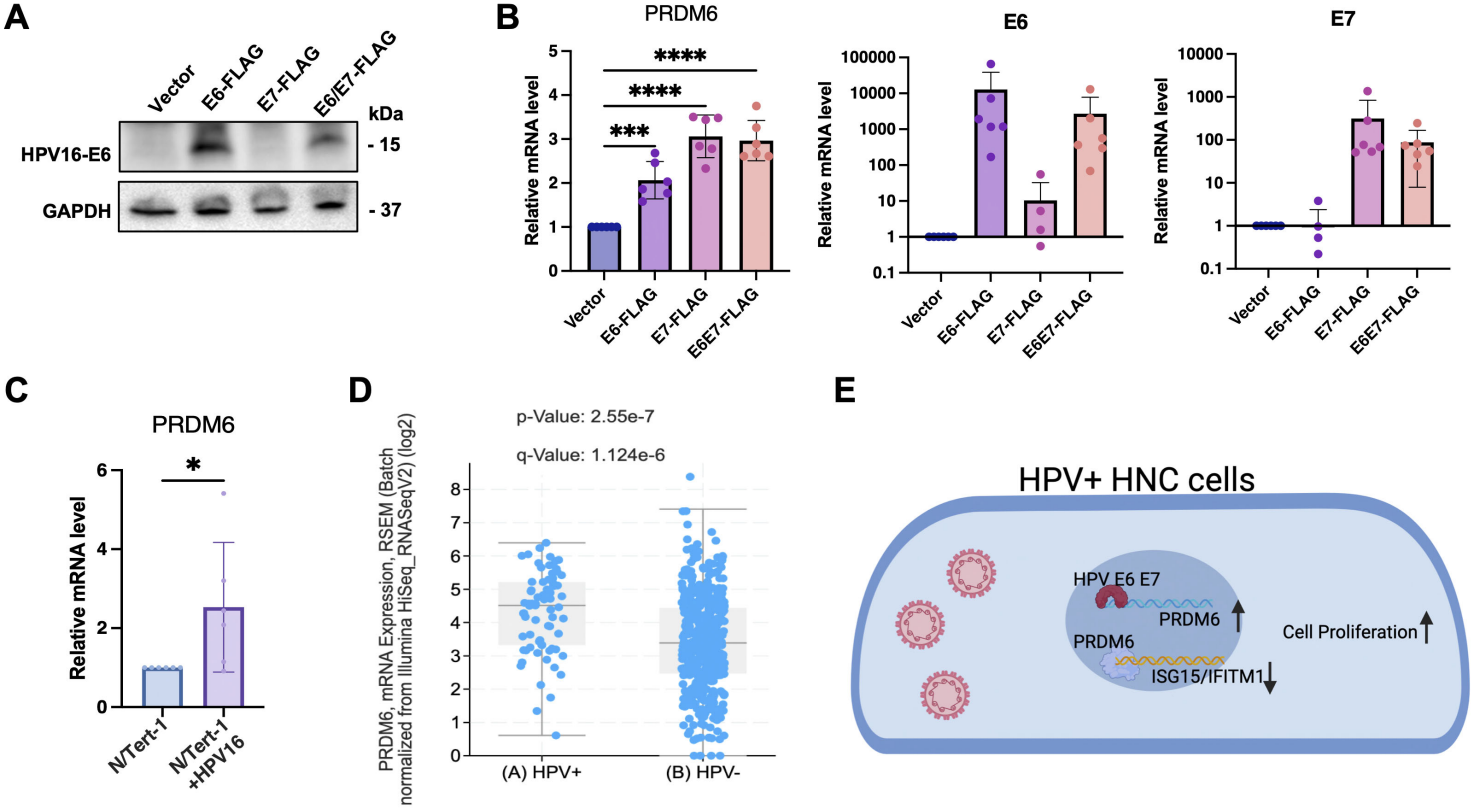
Induction of PRDM6 expression by HPV-16 E6/E7 viral oncoproteins in HNSCC tumor cells. **(A)** The pLXSN vector expressing FLAG-E6 (left panel), FLAG-E7 (middle panel), FLAG-E6/E7 (right panel), or the empty vector (EV) was transfected in Cal27 cells. Protein expression levels were measured through immunoblot. **(B)** Total RNA was extracted and analyzed by RT-qPCR to measure PRDM6 or E6/E7 transcripts, with normalization to GAPDH. **(C)** The telomerase-immortalized normal human foreskin keratinocytes with or without HPV-16 genome, N/Tert-1+HPV16 or N/Tert-1 cells, were subjected to RNA extractions and RT-qPCR analysis of PRDM6 transcripts with normalization to GAPDH. **(D)** HNSCC bulk RNA-seq datasets from TCGA were re-analyzed. Expression levels of PRDM6 in HPV-positive and negative (HPV+/-) HNSCC patients were compared. **(E)** A working model described that HPV may induce PRDM6 expression by E6/E7 viral oncoproteins to suppress expression of anti-tumor ISGs and thus promote development of HNSCC. Results were calculated from three independent experiments and shown as mean ± SD. (*p < 0.05, **p < 0.01, ***p < 0.001, ****p < 0.0001, Student’s t test).

High-risk HPV (16 and 18) is a risk factor for HNSCC and specifically linked to oropharyngeal squamous cell carcinoma (OPSCC). HPV-encoded E6 and E7 (E6/E7) viral oncoproteins play a key role in driving the development and progression of HPV-associated cancers through various mechanisms (33–35). We next determined whether PRDM6 may participate in HPV-mediated oral tumorigenesis. We exogenously expressed E6 and/or E7 oncoproteins (**Figure 4A**) and found that it indeed significantly increased PRDM6 expression in CAL27 cells, as measured by RT-qPCR analysis (**Figure 4B**). We further confirmed this finding in the telomerase-immortalized normal human foreskin keratinocytes (N/Tert-1) that harbor the HPV16 genome (N/Tert-1+HPV16), showing that PRDM6 expression is higher in N/Tert-1+HPV16 compared to the HPV16-native N/Tert-1cells (**Figure 4C**). Consistently, analysis of bulk RNA-seq from TCGA datasets revealed that PRDM6 expression elevates in HPV-positive HNSCC compared to HPV-negative cases (**Figure 4D**), while expression of ISGs (ISG15, IFITM1) exhibits the opposite trend (**Supplementary Figure S4**). Overall, our findings implicate that HPV may target PRDM6 to suppress immune gene expression through induction of its expression by E6/E7 viral oncoproteins, thus promoting oral tumorigenesis (**Figure 4E**).

## Discussion

In this study, we integrated multiple datasets of HNSCC scRNA-seq with the aim to identify tumor cell-specific transcriptional/epigenetic regulators that control immune gene expression and tumor cell intrinsic immunity. Such efforts led to the identification of 639 tumor-specific TF regulons, including 88 IRG-enriched ones. Among them, the histone methyltransferase PRDM6 emerged as a top, previously un-investigated TF that may regulate the immune response in HNSCC tumor cells. We demonstrated that PRDM6 expression occurs almost exclusively in tumor cells of clinical HNSCC tissues and that PRDM6 promotes proliferation and growth of HNSCC tumor cells *in vitro* while suppressing expression of anti-tumor ISGs (ISG15, IFITM1) by both loss- and gain-of-function approaches. We further identified that HPV E6/E7 oncoproteins upregulate PRDM6 expression in HNSCC tumor cells, which may link PRDM6 to HPV-induced oral oncogenesis.

TF gene regulatory networks as well as differentially expressed IRGs have been identified through analysis of HNSCC bulk RNA-seq datasets from TCGA (36–38). However, inherent limitations of bulk RNA-seq forbid the high-resolution characterizations of cellular heterogeneity within the tumor microenvironment, as gene expression levels across diverse types of cells in the tumor tissues are quantified with the setting that all types of cells are mixed together. Additionally, the regulon predictions primarily rely on the previous knowledge of benchmarked connections between TFs and their gene targets (39, 40). On the contrary, the scRNA-seq based transcriptomic profiling overcomes these limitations of bulk RNA-seq and enables the precise measurements of gene expressions that occur exclusively in malignant cells *vs* other types of cells in the tumor tissues, which permits us to annotate the HNSCC tumor cell-specific TF-IRG regulatory networks with the higher accuracy. IRIS3, powered by its unique bi-clustering algorithm and *de novo* motif prediction, has demonstrated its capability to identify the cell type-specific regulons with the satisfying reproducibility and robustness (16), which enabled our successful identifications of 88 IRG-enriched TFs in the HNSCC tumor cells (**Figure 1**).

We further characterized the histone methyltransferase PRDM6 as a potential TF that governs HNSCC tumor cell intrinsic immunity. Beyond the mere data mining, we experimentally verified that PRDM6 expresses in both HNSCC cancer cell lines and HNSCC clinic TMAs from multiple subjects (**Figure 2**). We also demonstrated PRMD6’s functions to promote proliferation and growth of HNSCC tumor cells, likely due to its activities to suppress expression of anti-tumor ISGs (**Figure 3**). These results are overall consistent with the early findings that PRDM family of proteins possess the oncogenic potency and regulate the tumor cell proliferation and differentiation (18, 19, 25, 41, 42). However, the precise mechanisms by which PRDM6 drives development and progression of HNSCC remain to be fully elucidated. Nevertheless, it has been showcased that PRDM6 targets the histone marks, including H3K27, in medulloblastoma (19). Our own analysis also confirms that PRDM6 regulates histone H3K27 trimethylation (H3K27me3). It is thus plausible that PRDM6-induced H3K27 trimethylation (H3K27me3) likely leads to the suppression of immune gene expression in tumor cells. Although the full spectrum of PRDM6-mediated methylation remains to be defined, this activity suggests a potential role in epigenetically regulating gene expression, including immune-responsive genes.

Our study also reveals that PRDM6 suppresses immune gene expression in HNSCC tumor cells, aligning with previous findings on the immunosuppressive roles of other PRDM family members. Notably, PRDM1 has been shown to modulate the tumor microenvironment by dampening immune responses, and its deletion has been associated with enhanced antitumor T cell activity (43). While the immunomodulatory function of PRDM6 remains less well characterized, our data indicate that PRDM6 upregulation attenuates type I interferon signaling, as evidenced by reduced expression of key interferon-stimulated genes (ISGs), including ISG15 and IFITM1. This repression may impair tumor immunosurveillance and promote tumor cell proliferation, both of which warrant further investigation. Additionally, PRDM6 has been reported to interact with the histone methyltransferase G9a, a known epigenetic regulator of inflammatory pathways and T cell function (44), suggesting a potential mechanism through which PRDM6 exerts immunosuppressive effects. Collectively, our findings suggest that PRDM6-mediated repression of anti-tumor ISGs may play a contributory role in oral tumorigenesis.

HPV highly associates with HNSCC, especially OPSCC, as nearly 80% of oropharyngeal cancers in the United States are infected with the high-risk HPV (16 and 18) (3). HPV E6/E7 viral oncoproteins are critical to drive malignant transformation of normal oral epithelial cells by targeting key cellular pathways with multiple strategies, such as degradation of p53 protein (45–47), activation of human telomerase reverse transcriptase (hTERT) (48, 49), and disruption of retinoblastoma (Rb) protein (50–52). Our results suggested a new link that HPV E6/E7 may manipulate PRDM6 expression to antagonize the antitumor and antiviral immune responses as a previously unappreciated viral mechanism to promote oral tumorigenesis (**Figure 4**). These findings would improve the fundamental understanding of HPV-associated HNSCC. However, it remains to be delineated how E6/E7 transcriptionally or epigenetically induces PRDM6 expression.

To summarize, our unbiased analysis of integrated HNSCC scRNA-seq datasets led to the identification of PRDM6 as a novel TF that governs the type I IFN signaling and immune gene expression and thus promotes proliferation and growth of tumor cells, while PRDM6 itself is also under control of HPV. These results would shed light in targeting PRDM6 for developing novel therapies to treat HNSCC. The potential immunosuppressive functions of PRDM6 would likely need to be interrupted or counteracted to boost antitumor immune responses, thus improving the efficacy of immunotherapies against HNSCC.

## Material and methods

### Cells

CAL27 and SCC9 cells were cultured in DMEM supplemented with 10% FBS. OKF6/TERT-2 (53) and TIGK cells (53) were both cultured in keratinocyte serum-free medium (K-sfm) with supplements (Thermo Scientific, CAT#17005042). Cell proliferation and growth was measured by using the ATP-based CellTiter-Glo Luminescent Cell Viability Assay (Promega, Cat. # G7572) following the manufacturer’s instructions and analyzed by the Cytation 5 multimode reader (luminescent mode).

### Quantitative PCR

Total RNAs were extracted using the NucleoSpin RNA extraction kit (Macherey-Nagel, Cat. # 740955.250) following the protocols provided by the manufacturer. RNA samples were reverse transcribed to cDNAs using iScript (BioRad, Cat. # 1708891). Real-time qPCR was performed on a CFX96 instrument (BioRad), by mixing 5 ul (2x) SYBR Green Supermix (BioRad, Cat. # 1725214), 0.5 ul primer mix, and 4.5 ul cDNA template. Data were analyzed by using the ΔΔCt method with GAPDH as an internal control. All qPCR primers were listed in **Supplementary Table S4**.

### Protein immunoblotting

Protein immunoblotting was performed as previously described (54). The following antibodies were used: anti-PRDM6 (Invitrogen, Cat. # PA5-43659), anti-GAPDH (Santa Cruz Biotechnology, Cat. # sc-47724), anti-mouse-HRP (Cell Signaling technology, Cat. # 7076), anti-rabbit-HRP (Cell Signaling technology, Cat. # 7074). The membrane was washed and blocked with 5% BSA, followed by the incubation with the primary antibody (1:1000 dilution) in 10 ml of the antibody dilution buffer for overnight at 4°C with shaking, and with the secondary anti-mouse or anti-rabbit antibody.

### Transfection and electroporation

Turbofect reagents (Thermo Scientific, Cat. # R0531) were used for plasmid transfection following the manufacturer’s recommendation as described previously (55). The pcDNA3.1 plasmid expressing the PRDM6 cDNA with FLAG tag at 3’ end was purchased from GenScript. The pLXSN plasmid expressing FLAG-tagged E6 (Cat. # 52395), E7 (Cat. # 52396), E6/E7 (Cat. # 52394) were acquired from Addgene. siRNAs targeting PRDM6, siPRDM6-1 (Cat. # s41097) and siPRDM6-2 (Cat. # s41098), were purchased from Invitrogen. For siRNA transfection in CAL27 and SCC9 cells, reverse transfection was performed using the Lipofectamine RNAiMAX (Invitrogen, Cat. # 13778100) as previously described (9).

### Protein immunofluorescence

Paraffin-embedded HNSCC tissue slides were baked at 65°C for 30 mins, followed by deparaffinization in xylene (10 mins × 2), 100% ethanol (10 mins × 2), 95% ethanol (5 mins), 70% ethanol (1 min), 50% ethanol (1 min), and finally rehydrated in double-distilled water (5 minus). Antigen unmasking was performed using 2100 Retriever (Electron Microscopy Sciences, Cat. # 62700-10) with the Antigen Retrieval Buffer (100X Tris-EDTA Buffer, pH9.0; Abcam, Cat. # ab93684) for 2 hrs to complete the cycle and then cool down. To block non-specific binding, slides were incubated with 10% normal goat serum (NGS) in PBST (0.1% Tween-20 in PBS) for 2 hrs at room temperature (RT). Incubation with the primary antibody was carried out overnight at 4°C using rabbit anti-PRDM6 antibody with 5% NGS in PBST. Sections were then washed with PBST and incubated with Alexa Fluor 488-conjugated, anti-rabbit secondary antibody with 5% NGS for 1 hr at RT. Nuclear counterstaining was performed using Hoechst 33342 (1:5000 in D-PBS) for 15 mins at RT. Coverslips were mounted on slides using ProLong Glass Antifade Mountant (Invitrogen, Cat. # P36982) and dried in the dark overnight. Confocal images were acquired using the ZEISS LSM 700 Upright laser scanning confocal microscope and processed with ZEN imaging software (ZEISS).

### Data analysis

Fastq files of the public bulk RNA-seq dataset (GSE243554) were obtained from GEO database and reanalyzed. Quality of raw reads was assessed by fastp (56). Reads were trimmed with adapters and aligned to the human genome (GRCh38.p14) with HISAT2 aligner. Uniquely aligned reads were submitted as inputs for mapping to gencode.v39 annotation. For identification of DEGs, DESeq2 was run with raw read counts obtained from FeatureCounts. Adjusted p value =< 0.05 and fold change >= 2 were used to filter for DEGs. For re-analysis of HNSCC scRNA-seq datasets, expression matrix was downloaded from GEO with the accession number GSE103322 (57), GSE150430 (58), GSE150321 (59), GSE162025 (60), and GSE150825 (61). Low-quality cells or empty droplets were filtered out based on the number of unique genes, the total number of molecules, and the percentage of mitochondrial reads detected in each cell. Normalization and scaling of cell clustering and visualization were performed using SeuratV4.0 (62). Cell identity was assigned based on the metadata and marker genes. Raw data of the public ChIP-seq datasets (GSE76496, GSE106058) were acquired from GEO and processed with ENCODE ChIP-seq-pipeline2. PRDM6 and H3K27me3 CUT&RUN peaks from GSE243557 were visualized using IGV. The code used for above analyses was deposited at github: https://github.com/ZhenyuWu-OSU.

### Statistics

Statistical analyses of experiment results were performed by using the unpaired, two-tailed Student’s *t* test or the one-way analysis of variance (ANOVA) in Graphpad PRISM 9.0 package. Variables were compared among all tested groups, and the P values below 0.05 were considered as statistically significant.

## Data Availability

The original contributions presented in the study are included in the article/**Supplementary Material**. Further inquiries can be directed to the corresponding authors.

## Supplementary material

Supplementary Figure 1
**(A)** Visualization of the TF-associated regulons for those TFs identified from at least 5 HNSCC patients. In total, 4,560 genes (blue) were predicted to under regulation of 24 TFs (orange), resulting in 36,696 predicted regulatory interactions between TFs and gene targets. TFs were clustered according to their eccentricity using Cytoscape. **(B)** Visualization of the TF-associated, IRG-enriched regulons for 24 TFs. The 4,560 genes were further filtered with the IRGs. In total, 1,636 IRGs (green) were predicted to under regulation of 24 TFs (red), resulting in 12,029 predicted regulatory interactions between TFs and IRGs. TFs were clustered according to their eccentricity using Cytoscape. **(C)** Pathway analysis was conducted for genes regulated by 24 TFs using ClusterProfiler, showing the enrichment of RNA processing pathway for general gene targets or viral response, transcription, and translation regulation pathways for IRGs, respectively.

Supplementary Figure 2
**(A–C)** Public PRDM6 ChIP-seq datasets GSE106058 **(A)** and GSE76496 **(B)**, as well as PRDM6 CUT&RUN dataset GSE243557 **(C)** were collected for re-analysis. The distribution of PRDM6 binding peaks across human genomes was visualized using Chipseeker. **(C)** Re-analysis of public PRDM6 ChIP-seq data (GSE76496) and H3K27me3 CUT&RUN data (GSE243557) demonstrated the similar distribution of PRDM6 and H3K27me3 bindings peaks. **(E, F)** Pathway analysis of the upregulated **(F)** and downregulated **(G)** gene expression in human neuroepithelial stem (NES) cells with PRDM6 overexpression (GSE243554).

Supplementary Figure 3 CAL27 cells were treated with IFN-α at the indicated doses. RNAs were extracted and subjected to RT-qPCR analysis of PRDM6 transcripts with normalization to GAPDH.

Supplementary Figure 4 HNSCC bulk RNA-seq datasets from TCGA were re-analyzed. Expression levels of ISG15 and IFITM1 in HPV+/- HNSCC patients were compared.

Supplementary Table 1 List of 639 TFs that regulate gene expression in HNSCC tumor cells.

Supplementary Table 2 List of 88 TFs that target IRG expression in HNSCC tumor cells identified from at least one patient.

Supplementary Table 3 List of 571 genes under regulation of PRDM6.

Supplementary Table 4 qPCR primers used in the study.
