# Supplementary figures and images for: Analysis of head and neck cancer scRNA-seq data identified PRDM6 promotes tumor progression by modulating immune gene expression
Source: Front Immunol. 2025 Aug 27;16:1596916. doi: 10.3389/fimmu.2025.1596916 (PMC12420620; doi:10.3389/fimmu.2025.1596916)

Figure S1

A

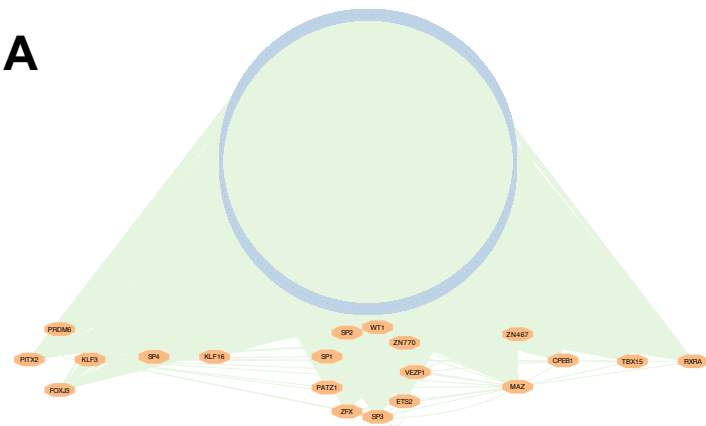

B

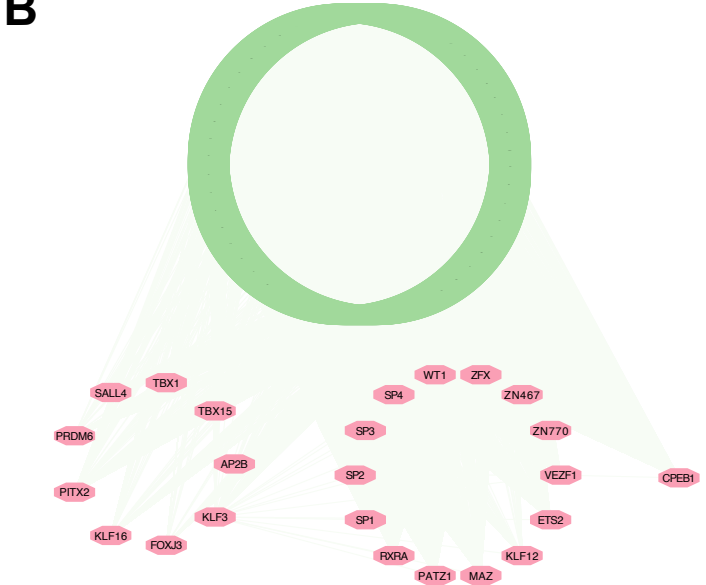

C

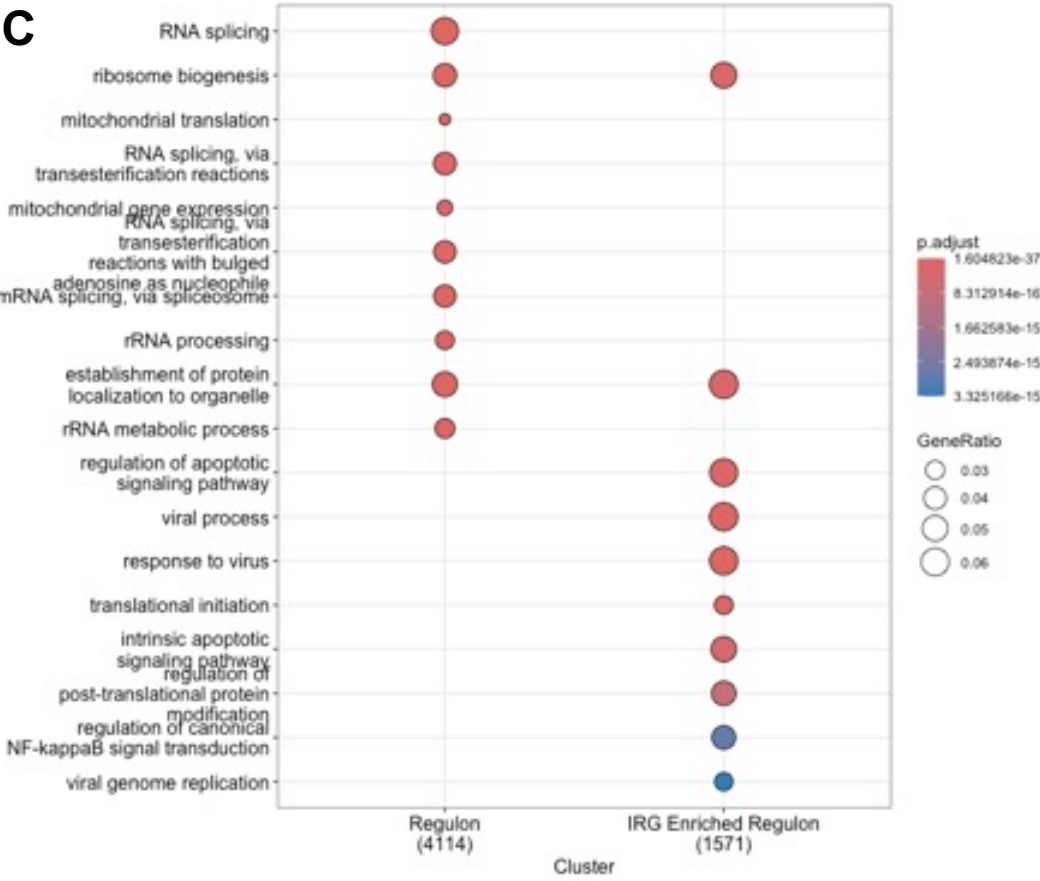

# Figure S2

**A**

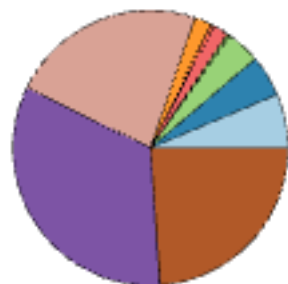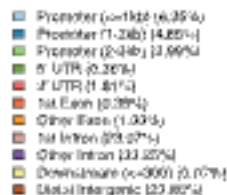

**B**

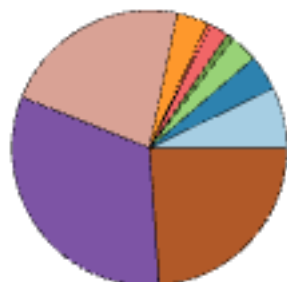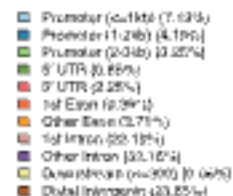

**C**

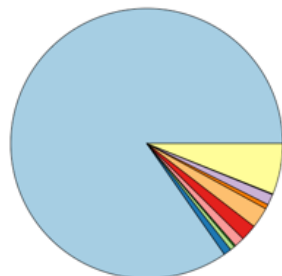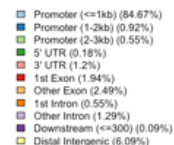

**D**

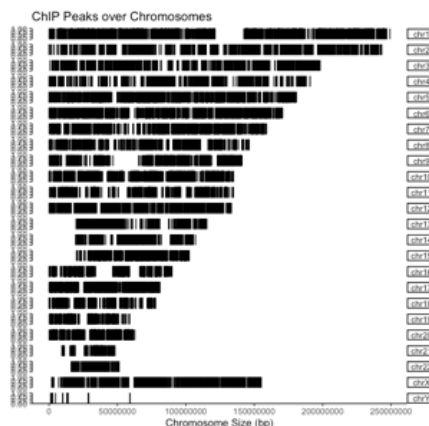

PRDM6

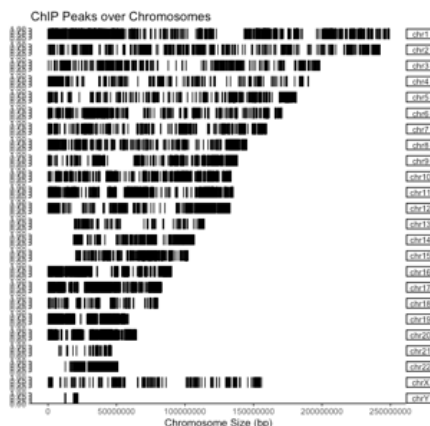

H3K27me3

**E**

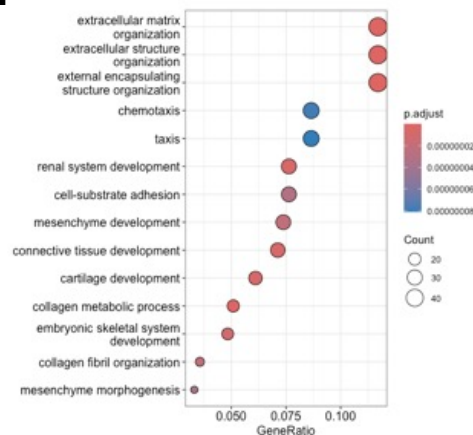

**F**

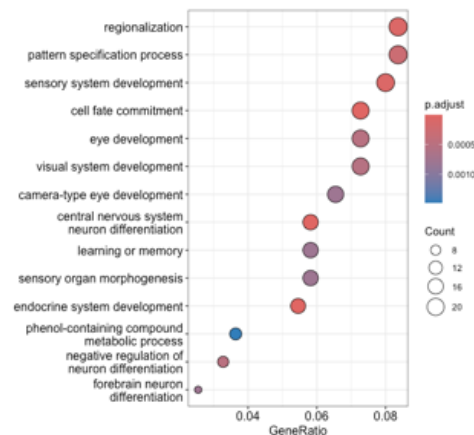

# Figure S3

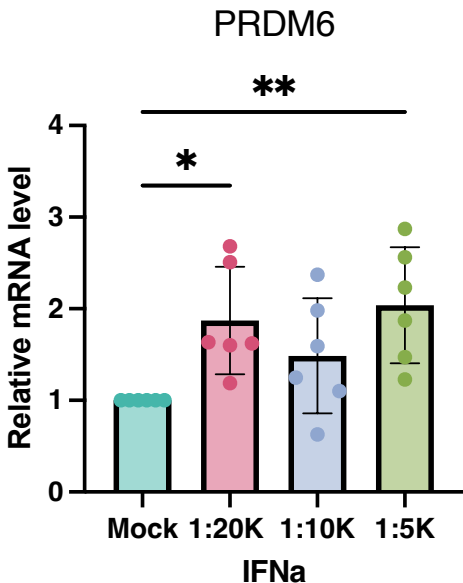

**Figure S4**

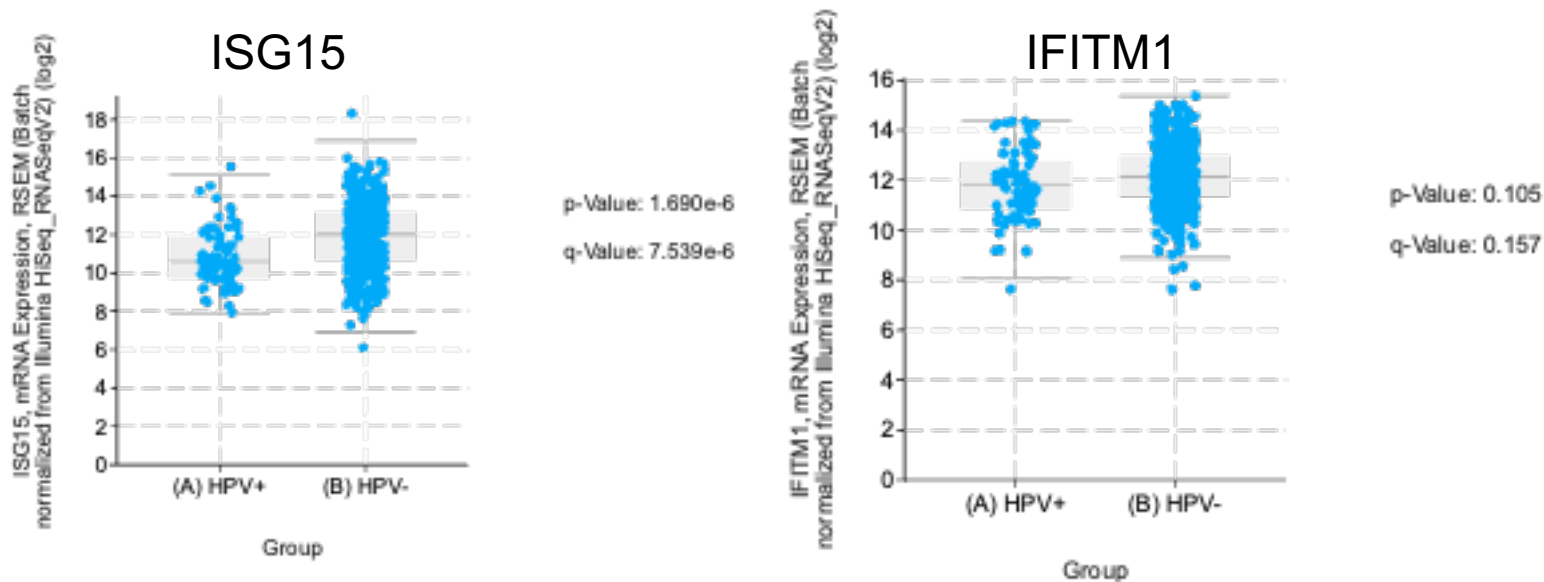

Supplement: Supplementary Figure 1 — (A) Visualization of the TF-associated regulons for those TFs identified from at least 5 HNSCC patients. In total, 4,560 genes (blue) were predicted to under regulation of 24 TFs (orange), resulting in 36,696 predicted regulatory interactions between TFs and gene targets. TFs were clustered according to their eccentricity using Cytoscape. (B) Visualization of the TF-associated, IRG-enriched regulons for 24 TFs. The 4,560 genes were further filtered with the IRGs. In total, 1,636 IRGs (green) were predicted to under regulation of 24 TFs (red), resulting in 12,029 predicted regulatory interactions between TFs and IRGs. TFs were clustered according to their eccentricity using Cytoscape. (C) Pathway analysis was conducted for genes regulated by 24 TFs using ClusterProfiler, showing the enrichment of RNA processing pathway for general gene targets or viral response, transcription, and translation regulation pathways for IRGs, respectively. [file DataSheet1.pdf]
